# Effect of Surface Treatments of Polyetherketoneketone as a Post Material on Shear Bond Strength to Root Dentin using Two Types of Resin Cement

**DOI:** 10.3290/j.jad.b3608791

**Published:** 2022-11-28

**Authors:** Masaaki Kasahara, Tomoko Someya, Masayuki Hattori

**Affiliations:** a Senior Assistant Professor, Department of Dental Materials Science, Tokyo Dental College, Misaki-cho, Kanda, Chiyoda-ku, Tokyo, Japan. Research idea, experimental design, hypothesis, and wrote the manuscript.; b Senior Assistant Professor, Department of Dental Materials Science, Tokyo Dental College, Misaki-cho, Kanda, Chiyoda-ku, Tokyo, Japan. Performed bond strength experiments, consulted on and performed statistical evaluation.; c Professor, Department of Dental Materials Science, Tokyo Dental College, Misaki-cho, Kanda, Chiyoda-ku, Tokyo, Japan. Interpreted and discussed the results, critical review of manuscript, and approved final manuscript.

**Keywords:** polyetherketoneketone (PEKK), post-and-core, root dentin, shear bond strength

## Abstract

**Purpose:**

This study investigated the effects of mechanical and chemical pretreatment of polyetherketoneketone (PEKK) on shear bond strength (SBS) to root dentin using two types of resin cement.

**Materials and Methods:**

A total of 100 PEKK specimens were prepared and polished. Sixty specimens were mechanically treated by sandblasting (50-µm Al_2_O_3_ for 10 s at 0.2–0.25 MPa), and the remaining 40 were untreated. Self-adhesive resin cement and conventional resin cement were used. PEKK specimens bonded to root dentin using self-adhesive resin cement were classified into three groups by pretreatment method: (1) untreated PEKK, (2) mechanical pretreatment (sandblasted PEKK), and (3) both mechanical and chemical pretreatment (PEKK sandblasting as well as application of Scotchbond Universal; 3M Oral Care). Conventional resin cement was pretreated following the same steps (1–3) as those followed for self-adhesive specimens. Each group included 10 specimens. PEKK specimens after surface treatments were examined using SEM. SBS tests were performed using a universal testing machine, and data were statistically analyzed using one-way ANOVA and Tukey’s multiple comparison test (p < 0.05).

**Results:**

No significant difference was observed between cements with and without sandblasting. However, self-adhesive specimens with both mechanical and chemical pretreatments demonstrated higher SBS than other cements with or without pretreatment.

**Conclusion:**

Mechanical pretreatment by sandblasting did not improve the PEKK-root dentin SBS. However, combined mechanical (sandblasting) and chemical pretreatment (ScotchBond Universal) significantly improved the SBS between the PEKK and root dentin.

The core buildup, which is used in endodontically treated teeth with major crown and pulp loss due to caries or injury, acts as an anchor for the crown prosthesis. Core buildup techniques commonly used in clinical settings are classified into two categories: (1) the integral structure of the post-and-core is built up by a monoblock casting technique and (2) the structure of the post-and-core is built up by combining a prefabricated metallic or glass-fiber post and a composite resin core. In the first technique, the metal for the core buildup, which has superior mechanical properties, can be easily customized according to the shapes of the root canal by the casting technique. However, because of a large difference in the elastic modulus between the metal used for the core buildup and dentin, excessive stress is concentrated on the post-dentin interface, possibling inducing severe root fracture.^5,11^ In contrast, the prefabricated glass-fiber post commonly used in the second technique has an elastic modulus somewhat closer to that of dentin compared with that of the metallic post, leading to a lower risk of root fracture.^4,21^ Nevertheless, the elastic modulus of the glass-fiber post (45.7–53.8 GPa^7^) is still more than twice that of dentin (20–25 GPa).^16^ Moreover, because glass-fiber posts are prefabricated, their ability to exactly conform to the oval root-canal cross-section is limited.^9^

Recently, particular attention has been paid to the application of polyaryletherketone (PAEK), a semi-crystalline thermoplastic resin.^13^ Polyetherketoneketone (PEKK) belongs the PAEK group, which has high biocompatibility and superior mechanical properties, and is used in crown restorative materials or dental prostheses.^8,17^ PEKK may be customized according to the root canal shapes, unique to each patient, through various processing techniques such as milling or pressing. PEKK has a compressive strength (246 MPa) close to that of dentin (275–300 MPa), but its elastic modulus is lower (5.1 GPa) than that of dentin.^2,16^ PEKK thus has a high potential for long-term use, compared with conventional materials used in clinical settings, in terms of superior biocompatibility and mechanical properties, provided that it is custom-made into a post-and-core structure.

Using three-dimensional finite analysis, Lee et al^19^ assessed the biomechanical behavior and long-term resistance of PEKK as a post-and-core material against root fracture. Their study reported that PEKK has a more favorable stress distribution and a lower risk of root fracture than conventional post-and-core materials. However, PAEK materials have the disadvantages of chemical inertness, low surface energy, and resistance to surface modifiers, interfering with better bonding to the materials. Similarly, Lee et al^19^ observed debonding of post cement and suggested that crown fracture might ensue, since the PEKK post-and-core system transferred stresses to the cement-restorative crown interface. Accordingly, to use PEKK as a post material, both the surface treatment technique and adhesive cements should be appropriately selected to provide durable bonding. Adhesive resin cements used mainly for bonding posts can be broadly classified into conventional types that require primer pretreatment and self-adhesive types that do not require pretreatment. Studies have discussed an improvement in adhesion when PEKK was used for the post material. However, these studies only reported cases where the adherend was not the root dentin,^29^ and pretreatment techniques and cement types were extremely limited.^14^

Against this background, this study discussed the effects of self-adhesive resin cements and conventional adhesive resin cements requiring pretreatment and the effects of mechanical and chemical pretreatments on the shear bond strength (SBS) at the PEKK/root-dentin interface. The null hypothesis was that the SBS of PEKK to root dentin would be equivalent even with different cements and surface treatment techniques.

## MATERIALS AND METHODS

### Preparation of Shear Bond Strength Test Specimens

**Fig 1:**
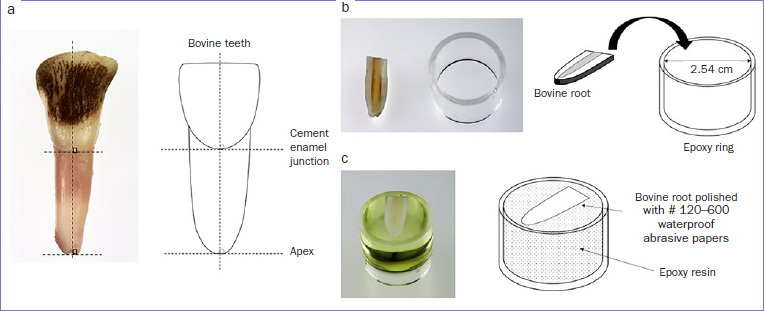
Schematic diagram and photos of specimen preparation used in the shear bond strength test. a. The bovine anterior tooth was split into a crown and a root at the cementoenamel junction, and the root was cut in half along its long axis. b. The bovine root cut in half was placed into an epoxy resin ring with the cut plane facing the top of the ring and embedded with epoxy resin (Scandiplex, Fritsch). c. The embedded specimen was polished with a 120-grit waterproof abrasive paper until the surface of the root dentin flattened and was then further polished with abrasive papers up to 600 grit.

Figure 1 shows a schematic diagram of test specimen preparation for the SBS test. In this study, 100 bovine anterior teeth were used. Frozen anterior teeth were thawed, and soft tissues still attached to the surfaces of the roots were removed. First, the bovine anterior tooth was split into a crown and a root portion at the cementoenamel junction, the tooth pulp was removed from the root canal, and the root was cut in half along its long axis (Fig 1a). Second, the bovine root half was placed into a 2.54-cm epoxy resin ring, with the cut plane facing the top of the ring, and embedded in epoxy resin (Scandiplex, Fritsch; Hagen, Germany; Fig 1b). The root was polished with a 120-grit waterproof abrasive paper until the surface of the root dentin was flattened, then further polished with abrasive papers up to 600 grit (Fig 1c). The dentin surface was treated with 18% ethylenediaminetetraacetic acid solution (Ultradent EDTA18%, Ultradent; South Jordan, UT, USA) for 20 s and then washed in distilled water. The root was dried with air. All treated specimens were stored in a moist environment at 37°C and 95% humidity for 1 week; thereafter, the specimens were taken out and washed in distilled water.

Table 1 lists the materials used in this study and their composition. A 12-mm-diameter Pekkton ivory press ingot (Pekkton, Cendres + Metaux SA; Biel/Bienne, Switzerland), as the PEKK adherend was cut into 1-mm-thick slices using a blade with a thickness of 300 μm (Serge Microtome, SP1600, Leica; Wetzlar, Germany) to prepare 100 specimens. The prepared specimens were polished with waterproof abrasive papers up to 600 grit. Then, the specimens were ultrasonically cleaned and left to air dry. Of the 100 specimens, 60 were mechanically pretreated by sandblasting with 50-µm Al_2_O_3_ at a pressure of 0.20–0.25 MPa for 10 s. The remaining 40 specimens were left untreated. Two self-adhesive resin cements (G-CEM [GCM, GC; Tokyo, Japan] and RelyX Unicem 2 AutoMix [UNA, 3M Oral Care; St Paul, MN, USA]) and two conventional resin cements requiring pretreatment (RelyX Ultimate Adhesive Resin Cement [ULR, 3M Oral Care] and Panavia V5 [PAF, Kuraray Noritake; Tokyo, Japan]) were used to create test specimens. Then, a double-sided tape with a 4-mm-diameter hole was affixed to the adhesive surface of the dentin to define the adhesive area.

**Table 1:** Details of the materials

Materials	Product (Lot No.)	Main components	Manufacturer	Code
Polyetherketoneketone (PEKK)	Pekkton ivory press ingot (0000351014)	Polyetherketoneketone, titanium dioxide	Cendres+Métaux (Biel/Bienne, Switzerland)	–
Adhesive	Scotchbond Universal (7647758)	10-MDP, dimethacrylate resins, HEMA, Vitrebond copolymer, filler, ethanol, water, initiators, silane	3M Oral Care (St Paul, MN, USA)	–
Self-adhesive resin cements	G-CEM (2007281)	Powder: fluoro-aluminosilicate glass, polymerization initiators, colorant Liquid: methacrylic ester, 4-AET, phosphoric acid ester monomer, water, silica, initiators	GC (Tokyo, Japan)	GCM
	RelyX Unicem2 AutoMix (7202815)	Base paste: glass powder, phosphoric ester monomer, TEG-DMA, silica, initiators Catalyst paste: glass powder, methacrylate, silica, initiators	3M Oral Care	UNA
Conventional resin cements requiring pretreatment	RelyX Ultimate Adhesive Resin Cement (7092708)	Base paste: glass powder, methacrylate, silica, initiators Catalyst paste: glass powder, methacrylate, silica, initiators	3M Oral Care	ULR
	Panavia V5 (4N0159)	Paste A: bis-GMA, TEG-DMA, hydrophobic aromatic dimethacrylate, hydrophilic aliphatic dimethacrylate, initiators, accelerators, silanated barium glass filler, silanated fluoro-aluminosilicate glass filler, colloidal silica Paste B: bis-GMA, hydrophobic aromatic dimethacrylate, hydrophilic aliphatic dimethacrylate, silanated barium glass filler, silanated aluminum oxide filler, accelerators, di-camphorquinone, pigments	Kuraray Noritake (Tokyo, Japan)	PAF


**Fig 2:**
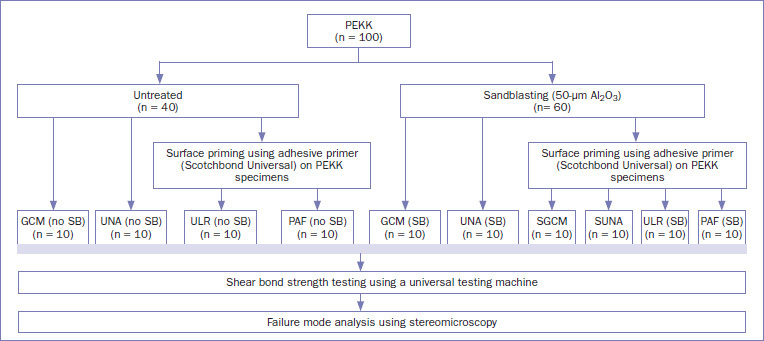
Flowchart of the experimental procedures.

Table 2 presents the various cements and pretreatments applied, and Fig 2 illustrates the experimental procedure used in this study. The self-adhesive cement specimens (GCM and UNA) were placed into 3 groups by pretreatment method: (1) untreated PEKK specimens bonded to dentin, (2) sandblasted PEKK specimens bonded to dentin, and (3) PEKK specimens (adherends) bonded to sandblasted and chemically-pretreated dentin. In the latter group, chemical pretreatment of dentin surfaces consisted of applying Scotchbond Universal (3M Oral Care) for 20 s, followed by air drying for 5 s. This was subsequently irradiated with light (BlueShot, Shofu; Kyoto, Japan) for 10 s before bonding. The conventional resin cements, ULR and PAF, requiring pretreatment were processed by following the same steps (1–3) as those for the self-adhesive cements (light irradiation was performed on ULR during treatment with Scotchbond Universal). To allow PEKK specimens to adhere to root dentin, adhesive resin cement was applied to the PEKK specimens, which were then manually pressed against the root dentin. After excessive cement was removed, it was irradiated with light from four directions for 10 s. The prepared specimens were stored in a moist environment at 37°C for 1 week.^22^ Each group included ten specimens.

**Table 2:** Surface treatment groups

Group	Procedure
GCM (no SB)*	**①** Untreated PEKK specimens, bonded to dentin using G-CEM
GCM (SB)*	**①** Sandblasting with 50-μm Al_2_O_3_ particles: treatment for 10 s at a pressure of 0.2–0.25 MPa on PEKK specimens **②** Bonded to dentin using G-CEM
S* GCM	**①** Sandblasting with 50-μm Al_2_O_3_ particles: treatment for 10 s at a pressure of 0.2–0.25 MPa on PEKK specimens **②** Priming using Scotchbond Universal on PEKK specimens and dentin (light cure for 10 s) **③** Bonded to dentin using G-CEM
UNA (no SB)	**①** Untreated PEKK specimens, bonded to dentin using RelyX Unicem2 AutoMix
UNA (SB)	**①** Sandblasting with 50-μm Al_2_O_3_ particles: treatment for 10 s at a pressure of 0.2–0.25 MPa on PEKK specimens **②** Bonded to dentin using RelyX Unicem2 AutoMix
SUNA	**①** Sandblasting with 50-μm Al_2_O_3_ particles: treatment for 10 s at a pressure of 0.2–0.25 MPa on PEKK specimens **②** Priming using Scotchbond Universal on PEKK specimen and dentin (light cure for 10 s) **③** Bonded to dentin using RelyX Unicem2 AutoMix
ULR (no SB)	**①** Priming using Scotchbond Universal on PEKK specimens and dentin **②** Bonded to dentin using RelyX Ultimate Adhesive Resin Cement
ULR (SB)	**①** Sandblasting with 50-μm Al_2_O_3_ particles: treatment for 10 s at a pressure of 0.2–0.25 MPa on PEKK specimens **②** Priming using Scotchbond Universal on PEKK specimen and dentin **③** Bonded to dentin through RelyX Ultimate Adhesive Resin Cement
PAF (no SB)	**①** Priming using Scotchbond Universal on PEKK specimens and dentin (ight cure for 10 s) **②** Bonded to dentin using Panavia V5
PAF (SB)	**①** Sandblasting with 50-μm Al_2_O_3_ particles: treatment for 10 s at a pressure of 0.2–0.25 MPa on PEKK specimens **②** Priming using Scotchbond Universal on PEKK specimens and dentin (light cure for 10 s) **③** Bonded to dentin using Panavia V5

No SB: no sandblasting; SB: sandblasting; S: surface priming using adhesive primer (Scotchbond Universal).

### Surface Observations

In addition to the SBS test specimens, two PEKK specimens were prepared for each of the three groups: untreated PEKK specimens, sandblasted PEKK specimens, and PEKK specimens mechanically treated by sandblasting and chemically treated with a universal adhesive. The surfaces of the PEKK specimens were observed under a field-emission scanning electron microscope (FE-SEM´ SU6600, Hitachi; Tokyo, Japan). The specimens were Au-Pd sputter-coated before FE-SEM observation. The elemental composition of the specimen surfaces was analyzed using energy-dispersive x-ray spectroscopy (EDX) at a 10-mm working distance and 15.0-kV operating voltage.

### Shear Bond Strength Test

**Fig 3:**
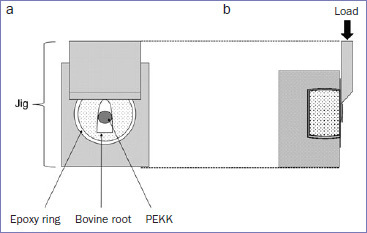
Schematic diagram of mounting the specimen on the jig of the shear bond strength testing machine. The specimen was mounted with its root apex side facing upward in the jig of the testing machine, and shear force was applied in the direction from the root apex toward the crown. a. Front view of the jig with the mounted specimen; b. side view of the jig with the mounted specimen.

After 1 week of storage, the test specimens were removed from the moist environment and mounted on the SBS testing jig (Fig 3) of a universal testing machine (EZ Graph, Shimadzu; Kyoto, Japan). Shear force was applied at a crosshead speed of 0.5 mm/min from the root apex toward the crown. SBS was calculated from the obtained maximum fracture loads. After testing, the fracture surfaces were observed at 2.5X magnifcation under a stereomicroscope equipped with a digital camera (Stemi 508, Zeiss; Oberkochen, Germany).

### Compressive Strength Test

To verify the mechanical strength of the cements used in this study, the compressive strength test was performed on individual specimens created by injecting the respective cement into 4.0-mm (diameter) x 8.0-mm (height) transparent acrylic tubes. These were light irradiated perpendicular to the long axis of the specimen for a period specified by the manufacturer. After curing, the acrylic tubes were removed from the resulting cylindrical specimens. The specimens were then stored in a moist environment at 37°C for 1 week. After 1 week of storage, compressive strength was tested in a universal testing machine (EZ Graph, Shimadzu) at a crosshead speed of 0.5 mm/min. The loads at fracture were recorded and the compressive strength was calculated from these.

### Statistical Analysis

One-way ANOVA was performed to determine whether the mean SBS and the compressive strength of each specimen differed significantly, followed by Tukey’s multiple comparisons test (SPSS v 25, IBM; Armonk, NY, USA). Statistical significance was set at p < 0.05.

## RESULTS

### Observations of Treated Surfaces

**Fig 4:**
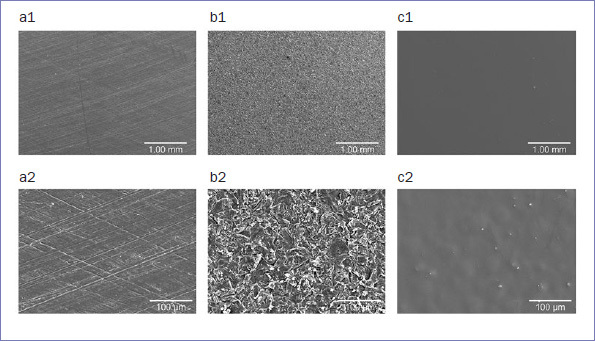
SEM images of polyetherketoneketone surfaces after different surface treatments. a. No treatment (a1, magnification 30X; a2, magnification 300X); b. Sandblasting with 50-µm Al_2_O_3_ particles (b1, magnification 30X; b2, magnification 300X); c. Sandblasting with 50-µm Al_2_O_3_ particles and priming using Scotchbond Universal (c1, magnification 30X; c2, magnification 300X).

Figure 4 shows the surfaces of the untreated PEKK specimens (a1, a2), sandblasted specimens (b1, b2), and specimens sandblasted and primed using Scotch Bond Universal Adhesive (c1, c2). The surfaces of the untreated PEKK specimens (a1, a2) were relatively smooth and even, except for a few tool marks. The sandblasted PEKK specimens (b1, b2) had irregular, rough surfaces. The surfaces of the PEKK specimens that were sandblasted and primed using Scotch Bond Universal Adhesive (c1, c2) were relatively smooth, except for a slight unevenness because the sandblasted surfaces were covered with the priming agent.

**Fig 5:**
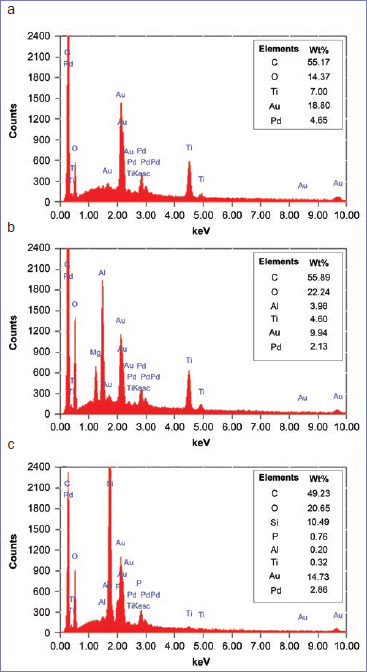
Energy-dispersive x-ray spectroscopy spectra of different surface treatments. a. No treatment; b. sandblasting with 50-µm Al_2_O_3_ particles; c. sandblasting with 50-µm Al_2_O_3_ particles plus priming using Scotchbond Universal.

Figure 5 shows the EDX spectra and element compositions of the surfaces of the PEKK specimens obtained. C, O, and Ti were detected on the surfaces of the untreated PEKK specimens (a). Al was detected on the surfaces of the PEKK specimens sandblasted with Al_2_O_3_ (b). C, O, Si, and small amounts of Al and P were found on the surfaces of the PEKK specimens sandblasted and primed using Scotch Bond Universal (c).

### Shear Bond Strength

One-way ANOVA showed a significant difference in SBS between the groups (p < 0.05). Table 3 shows the SBS at the PEKK/root-dentin interfaces bonded with different adhesive resin cements. No significant difference in SBS was observed between the cement products used in the untreated and sandblasted PEKK specimens. SBS was higher in PEKK specimens with conventional ULR than in some untreated PEKK specimens with self-adhesive cement (p < 0.05). In contrast, SBS was higher in PEKK specimens with self-adhesive cements, which were both mechanically and chemically pretreated, than that of PEKK specimens with other cements and pretreated by other techniques (p < 0.05).

**Table 3:** Comparison of the mean ± SD shear bond strengths (MPa) between cements

Group		Shear bond strength
GCM	no SB SB SGCM	2.3 ± 1.2^d^ 3.5 ± 0.9^cd^ 10.4 ± 4.2^ab^
UNA	no SB SB SUNA	3.9 ± 2.4^cd^ 3.3 ± 1.5^cd^ 11.9 ± 4.8^a^
ULR	no SB SB	6.9 ± 4.5^bc^ 7.4 ± 3.7^bc^
PAF	no SB SB	3.7 ± 1.8^cd^ 4.5 ± 2.0^cd^

GCM (G-CEM): no SB, untreated PEKK specimens, bonded to dentin using G-CEM; SB: sandblasted with 50-μm Al_2_O_3_ particles; SGCM: sandblasted with 50-μm Al_2_O_3_ particles, primed using Scotchbond Universal on PEKK specimens and dentin; UNA (RelyX Unicem 2 AutoMix): no SB, untreated PEKK specimens bonded to dentin using RelyX Unicem 2 AutoMix; SB: sandblasted with 50-μm Al_2_O_3_ particles; SUNA: sandblasted with 50-μm Al_2_O_3_ particles andprimed using ScotchBond Universal on the PEKK specimens and dentin; ULR (RelyX Ultimate Adhesive Resin Cement): no SB, untreated PEKK specimens bonded to dentin using RelyX Ultimate Adhesive Resin Cement; SB: sandblasted with 50-μm Al_2_O_3_ particles. PAF (Panavia V5): no SB, untreated PEKK specimens bonded to dentin using Panavia V5; SB: sandblasted with 50-μm Al_2_O_3_ particles. Different superscript letters indicate significant differences between the experimental groups (p < 0.05).

The modes of failure of the PEKK specimens after testing are shown in Table 4. Regardless of the cement type, many adhesive failures were observed at the PEKK-cement interfaces in all untreated specimens. SUNA (priming using Scotchbond Universal on sandblasted PEKK specimens and root dentin, then bonded to PEKK and root dentin with RelyX Unicem2 AutoMix), which had the highest bond strength, showed no adhesive failures between PEKK and cement, indicating either cement-dentin adhesive failure or mixed failure.

**Table 4:** Failure mode distribution

Group		Failure between dentin and cement (n)	Failure between PEKK and cement (n)	Mixed adhesive and cohesive failure within cement (n)
GCM	no SB SB SGCM	5	5	0
		4	1	5
		1	3	6
UNA	no SB SB SUNA	2	8	0
		4	0	6
		7	0	3
ULR	no SB SB	2	8	0
		5	2	3
PAF	no SB SB	0	9	1
		3	3	4

GCM (G-CEM): no SB, untreated PEKK specimens bonded to dentin using G-CEM; SB: sandblasted with 50-μm Al_2_O_3_ particles; SGCM, sandblasted with 50-μm Al_2_O_3_ particles and primed using Scotchbond Universal on the PEKK specimens and dentin; UNA (RelyX Unicem 2 AutoMix): no SB, untreated PEKK specimens bonded to dentin using RelyX Unicem 2 AutoMix; SB: sandblasted with 50-μm Al_2_O_3_ particles; SUNA: sandblasted with 50-μm Al_2_O_3_ particles and primed using ScotchBond Universal on the PEKK specimens and dentin; ULR (RelyX Ultimate Adhesive Resin Cement): no SB, untreated PEKK specimens bonded to dentin using RelyX Ultimate Adhesive Resin Cement; SB: sandblasted with 50-μm Al_2_O_3_ particles. PAF (Panavia V5): no SB, untreated PEKK specimens bonded to dentin using Panavia V5; SB: sandblasted with 50-μm Al_2_O_3_ particles.

### Compressive Strength

Table 5 shows the compressive strength of the individual cement products. The mean compressive strengths ranged from 246 to 272 MPa, and no significant difference was observed between the cements (p > 0.05).

**Table 5:** Compressive strengths (MPa) of cements used in this study

Group	Compressive strength
GCM	245.8 ± 48.0
UNA	252.6 ± 44.9
ULR	259.7 ± 30.1
PAF	271.8 ± 38.8

GCM: G-CEM; UNA: RelyX Unicem 2 AutoMix; ULR: RelyX Ultimate Adhesive Resin Cement; PAF: Panavia V5. No significant differences were observed between the cements.

## DISCUSSION

PEKK has the potential to be an attractive post-and-core material because of its adequate mechanical strength and shock-absorption properties, as well as its ability to be customized through various processing methods. A previous study^19^ that evaluated the biomechanical behavior and long-term performance of PEKK post-and-core, metal, and fiber-post/resin-core using the finite element method found that PEKK, which has the lowest elastic modulus, was the least likely to cause root fracture. On the other hand, PEKK post-and-core systems showed more debonding under long-term cyclic loading than did metal or fiberglass post-and-core systems. This indicates that strong adhesion to root canal dentin is important for the long-term performance of PEKK posts.

This study showed that both treatment techniques – mechanical treatment by sandblasting and chemical treatment with Scotchbond Universal – might improve the bond strength at the PEKK-dentin interface. Thus, the null hypothesis was rejected.

### Pretreatment of PEKK Specimens

Various types of mechanical and chemical treatment techniques have been experimentally applied to PEKK specimens. PEKK possesses chemical inertness, low surface energy, and resistance to surface modification, as do other types of PAEK materials, which allow it to achieve durable bonding at the interface between the resin and PEKK materials.^10,18,20,25,29,30,33^ One study reported that chemical treatment of PEKK with H_2_SO_4_ improved bond strength.^18^ However, PEKK surface treatment with H_2_SO_4_ at chairside may harm the patient. In contrast, mechanical pretreatment by sandblasting using Al_2_O_3_ particles is common in clinical settings, and some studies have reported that this technique improved the bond strength of PEKK.^10,13,14,18,20,29,30,33^

The SEM observations in this study revealed irregular, rough surfaces on PEKK specimens. Failure mode analysis showed that the sandblasted PEKK group had fewer adhesive failures between PEKK and cement than did the untreated group. This suggests that mechanical pretreatment by sandblasting resulted in a certain degree of mechanical retention.

However, no significant difference in SBS was observed between any cement group pretreated by sandblasting vs the untreated cement experimental groups. Stawarczyk et al^30^ reported that when PEKK was mechanically pretreated by sandblasting at a pressure of 0.2 MPa and chemically pretreated with a dimethacrylate pretreatment agent (PEKKbond, anaxdent North America; Ardmore, PA, USA), the tensile bond strength (TBS) was insufficient. These results indicated that obtaining a suitable PEKK surface morphology to yield high bond strength to resin cement may depend on the pressure of sandblasting. One study reported that sandblasting at a pressure of 0.2 MPa did not affect the bond strength to PEKK,^25^ whereas other studies reported that sandblasting at a pressure 0.5 MPa improved the bond strength to PEKK.^18,20^ Sandblasting in this study was performed at 0.2–0.25 MPa. These pressures are relatively common when sandblasting fiber posts.^1,26^ However, it was suggested that this did not contribute to the improvement of the bond strength to PEKK.

Stawarczyk et al^30^ used a methacrylate pretreatment agent, Visio.Link (Bredent; Senden, Germany), and observed higher TBS in PEKK specimens chemically pretreated with Visio.Link than in PEKK specimens pretreated with PEKKbond, even under the same sandblasting pressure of 0.2 MPa.^30^ They further suggested that the components of Visio.Link effectively dissolved the PEKK surfaces, improving the bond strength without being affected by the sandblasting pressure.

In this study, Scotchbond Universal was applied to PEKK specimens as chemical pretreatment. One study^20^ found that Scotchbond Universal showed higher bond strength to PEKK without mechanical and/or chemical pretreatment with sulfuric acid compared to other pretreatment materials used in another study on PEKK.^8^ Similarly, in this study, ULR chemically pretreated with Scotchbond Universal demonstrated higher SBS than did the self-adhesive GCM or UNA resin cements. Additionally, self-adhesive cements mechanically pretreated with sandblasting and chemically pretreated with Scotchbond Universal showed higher SBS than other types of cements pretreated using any other techniques. Scotchbond Universal contains silane and 10-methacryloyloxydecyl dihydrogen phosphate (10-MDP) monomer. Si and P were detected by EDX on the PEKK surfaces; this demonstrated that the surfaces of the PEKK specimens were appropriately chemically pretreated. Pretreatment materials containing 10-MDP monomer were reported to have bond strengths similar to those of methacrylate pretreatment materials,^13^ which are known to provide high bond strengths to PAEK materials.^30^ To verify the finding, the present authors conducted an additional experiment. PEKK specimens were pretreated with a 10-MDP-containing pretreatment agent (Panavia V5 Tooth Primer, Kuraray Noritake) or a silane-containing pretreatment agent (Porcelain Primer, Shofu), and the SBS to UNA was measured, as it showed the highest bond strength in this study (n = 10). Higher bond strength was observed to PEKK specimens pretreated with 10-MDP and silane monomers than in untreated PEKK specimens (Table 6). This suggests that not only 10-MDP monomer but also silane monomer contributes to the bond strength of the surfaces of PEKK specimens.

**Table 6:** Additional experiments: Comparison of the mean ± SD shear bond strengths (MPa) among pretreatment materials

Group	Shear bond strength
UNA (10-MDP)	9.5 ± 1.5
UNA (silane)	8.1 ± 2.6

UNA (10-MDP): PEKK specimens (sandblasted with 50-μm Al_2_O_3_ particles) were pretreated with a material containing 10-MDP monomer (Panavia V5 Tooth Primer, Kuraray Noritake) bonded to dentin using UNA (RelyX Unicem 2 AutoMix). UNA (silane): the PEKK specimens (sandblasted with 50-μm Al_2_O_3_ particles) were pretreated with a material containing silane monomer (Porcelain Primer, Shofu), then bonded to dentin using UNA (RelyX Unicem 2 AutoMix). No significant differences were observed between the cements.

The results of EDS analysis showed Ti (originating from PEKK) and Al (originating from Al_2_O_3_) on the PEKK surface. The chemical bonding to Ti and Al of the phosphate ester groups of 10-MDP and the silanol groups produced by hydrolysis of silane coupling agents may have contributed to the improvement in bond strength. PEKK is chemically stable but hydrophobic.^24^ Therefore, PEEK may have a high affinity for hydrophobic structures such as methacryloyloxy groups in the pretreatment agents. However, since several monomers with these functional groups are contained in each pretreatment, further research is needed to determine which mechanism contributes to the improvement in bond strength.

### Bond Strength of PEKK to Root Dentin

To the best of our knowledge, only Wang et al^33^ investigated the SBS at the PEKK-dentin interface. They focused on the bond strength of a dental prosthesis to crown dentin.^33^ In this study, root dentin was used as the adherend to investigate the applicability of adhesive resin cement to post material. Root dentin has fewer exposed dentinal tubules than does crown dentin, suggesting that the bond strength decreases.^23^ Several studies have reported the bond strengths to dentin of several adhesive resin cements.^3,6,7,15,31,32^ Many of these studies found that the adhesive resin cements requiring pretreatment had a slightly higher bond strength than did self-adhesive resin cements.^6,12,31^ Similarly, in this study, pretreated ULR and PAF had slightly higher bond strengths. This finding is in agreement with the results of the study by Someya et al^28^ on the SBS between dentin and adhesive resin cement. They assumed that self-adhesive cements take time to fully cure, which affects their mechanical properties. However, no significant difference in compressive strength was observed between the cements stored under the same conditions and used in this study. In contrast, Someya et al^28^ also performed a pull-out test as well as a SBS test on the root dentin using a fiber post and reported lower retention of self-adhesive cements compared to those requiring pretreatment. According to those authors, the post had a low retentive force because resin tags were not fully formed in root dentin by the self-adhesive resin cement. The same findings were reported previously.^27^ In the present study, fracture surface observations demonstrated that when PEKK specimens were pretreated appropriately, adhesive failure was observed in relatively large numbers at the cement-dentin interface. As with previous studies, resin tags were probably not fully formed in this study.

## CONCLUSION

This study assessed the effects of mechanical and chemical surface pretreatment and the effects of different resin cements on the SBS at the PEKK-root dentin interface. The SBS at the PEKK-root dentin interface did not improve with mechanical pretreatment by sandblasting with 50-μm Al_2_O_3_ at a pressure of 0.20-0.25 MPa for 10 s. However, the SBS at the PEKK-root dentin interface significantly improved with a combination of sandblasting and chemical pretreatment with 10-MDP and silane monomers. Thus, PEKK prepared with these pretreatment techniques has potential for use as a post material. However, further studies are warranted to determine the optimal sandblasing pressure for PEKK and its affinity for chemical solvents. In the future, studies should be conducted involving thermocycling and fatigue tests to investigate the long-term potential for retention on dentin.

## Clinical relevance:

To assess the possibility of using PEKK as a post material, mechanical pretreatment by sandblasting and chemical pretreatment with 10-MDP and silane was performed on PEKK specimens. These pretreatments improved the PEKK-root dentin shear bond strength.
